# Incidence of portosystemic shunts in Finnish miniature schnauzer litters

**DOI:** 10.1186/s13028-026-00858-5

**Published:** 2026-02-22

**Authors:** Jenni Maria Sukura, Thomas Spillmann, Outi Maria Laitinen-Vapaavuori, Sari Helena Mölsä

**Affiliations:** Department of Equine and Small Animal Medicine, Faculty of Veterinary Medicine, University of Helsinki, P.O. Box 57, Helsinki, FI-00014 Finland

**Keywords:** Bile acid, CPSS, Dog, Hepatic anomaly, Liver, Vascular anomaly

## Abstract

**Background:**

Congenital portosystemic shunts (CPSSs) are vascular anomalies that permit communication between the portal and systemic circulation, thus allowing venous blood to bypass the liver. Previous studies have reported an estimated CPSS prevalence of 0.18–0.76% in the general dog population, being more commonly diagnosed in purebred breeds such as cairn terriers, Maltese, dachshunds, Yorkshire terriers, Irish wolfhounds, golden retrievers and Labrador retrievers. In addition, miniature schnauzers are known to be predisposed to CPSSs, but the breed-specific incidence has not been systematically investigated. This study was undertaken to evaluate the incidence of CPSSs in Finnish 6–12-week-old miniature schnauzer puppies within a one-year study period. The puppies were screened for the presence of a CPSS by measuring the postprandial serum bile acid (SBA) concentration. When the SBA concentration was above the reference limit, further investigations were performed (i.e., pre- and postprandial bile acid stimulation test, computer tomography (CT) or post-mortem examination). Based on the results, the incidence of CPSSs was calculated.

**Results:**

The study included 582 dogs from 121 litters, representing 58% (582/1004) of the miniature schnauzers born and registered in Finland during the 1-year study period. In screening, an elevated postprandial serum SBA concentration was presented in 3.3% (19/582) of the dogs. In retesting with the SBA stimulation test 1–4 weeks later, 11 of these 19 dogs had pre- and postprandial bile acid concentrations within the reference range and were excluded from further investigations. One dog with elevated SBA concentrations was diagnosed with portal vein hypoplasia and seven dogs with a CPSS. In addition, one dog had a normal postprandial SBA concentration at screening but was later diagnosed with a CPSS as an incidental finding by CT. In summary, a CPSS was diagnosed in 8/582 dogs and the incidence was 1.37%. All diagnosed CPSS vessels originated either from the splenic vein (*n* = 5) or the left gastric vein (*n* = 3) and inserted into the caudal vena cava at the level of the diaphragm.

**Conclusions:**

The incidence of the congenital portosystemic shunts in Finnish miniature schnauzers was elevated when compared to the estimated prevalence in the general dog population. The morphology of the portosystemic shunts could indicate a common genetic background.

## Background

Congenital portosystemic shunts (CPSSs) are vascular anomalies of the liver that allow abnormal communication between the portal vein, or its tributaries, and the systemic circulation [1–6]. This condition enables venous blood from the gastrointestinal tract, spleen and pancreas to bypass the liver, leading to toxin accumulation in the bloodstream, liver parenchymal atrophy and hepatic insufficiency [1, 6].

CPSSs can be classified as intrahepatic (ICPSSs) or extrahepatic (ECPSSs), depending on the location of the abnormal vascular connection, and may involve either a single or multiple aberrant vessels [2, 5, 6]. The type of CPSS often correlates with the size of the dog [7, 8]. An extrahepatic CPSS (ECPSS) is most frequently observed in small dog breeds, with predisposition reported in cairn terriers, dachshunds, Yorkshire terriers, Maltese, miniature schnauzers, poodles and Jack Russell terriers [5–14].

A CPSS has significant clinical effects on the neurological, gastrointestinal, and urinary systems [1, 5, 6]. Elevated bile acids and/or ammonia levels combined with clinical findings such as hepatic encephalopathy, ammonium urate crystals or urolithiasis in a puppy strongly indicate a portosystemic shunt [5, 6, 15, 16]. The serum bile acid (SBA) stimulation test has demonstrated a maximum sensitivity of 100% for CPSSs, and some studies have reported 98–100% sensitivity with a single postprandial serum bile acid sample, making it an ideal screening tool [17, 18]. In contrast, the fasting ammonia concentration, although sensitive (85%) and specific (86%), is less practical due to the instability of ammonia [16, 19]. Diagnosis is typically confirmed by abdominal ultrasound and/or computed tomography (CT) [6, 20].

While a CPSS can often be treated surgically or conservatively [21, 22], it causes significant suffering for affected dogs and imposes substantial economic costs on owners. Therefore, preventing a CPSS or ensuring early diagnosis is critical for both animal welfare and responsible breeding practices.

A genetic basis for CPSSs is strongly suspected due to their higher prevalence in specific breeds. Genetic associations have been confirmed in Irish wolfhounds [23–25], cairn terriers [11], Maltese [14] and Yorkshire terriers [10]. The prevalence in the general dog population ranges from 0.18% to 0.76% [7, 13], but in high-risk breeds, it is estimated to be markedly higher, from 1.6 to 3.6% [7, 10, 23].

Miniature schnauzers are known to be predisposed to ECPSSs [7], but the breed-specific prevalence has not been systematically investigated. Interestingly, one study [12] reported that a CPSS in miniature schnauzers is often diagnosed later in life compared to other breeds. This suggests that the breed may exhibit milder cases or even cases without clinical signs, although the underlying reasons remain unclear. Possible explanations include morphological differences in the CPSS or other breed-specific factors affecting the expression of clinical signs [8, 20, 26, 27]. The number of CPSS cases diagnosed by veterinarians may not reflect the true prevalence of this disease.

The aim of this study was to evaluate the incidence of CPSSs in Finnish miniature schnauzers. The hypothesis was that miniature schnauzers have an elevated incidence of CPSSs compared to most other breeds.

## Methods

### Recruitment and inclusion criteria of the dogs

Ethical permission for this research was provided by the Finnish national Project Authorisation Board (ESAVI/8155/2020), and all owners signed a consent form. Between 18.02.2021 and 17.02.2022, 6–12-week-old miniature schnauzer puppies with a pedigree born in Finland were recruited into the study. The dogs were recruited in the order of enrolment through social media and by contacting breeders/owners through the miniature schnauzer breed club. All puppies registered for the litter were evaluated. If only a part of the litter participated, samples were excluded from the study.

### Evaluation of dogs

The study included a screening visit involving the measurement of postprandial SBA. Written instructions on how to prepare for and perform a postprandial SBA measurement for the study were sent in advance to the breeders and attending veterinarians. The breeders fasted the puppies for 6–8 h prior feeding a meal. They were instructed not to give low-fat food and to ascertain that all the puppies ate a normal portion of food. A venous blood sample for measurement of the postprandial bile acid concentration was taken from each puppy approximately 2 h after the meal. All the puppies were identified; both the microchip number and registration with the Finnish Kennel Club breed registry were checked. The litter size and possible abnormalities noticed by the attending veterinarian were recorded. Samples were taken either at the Veterinary Teaching Hospital of the University of Helsinki (VTHUH) or at cooperating veterinary hospitals or clinics around Finland.

### Interpretation of bile acid results and further evaluations

**Fig. 1:**
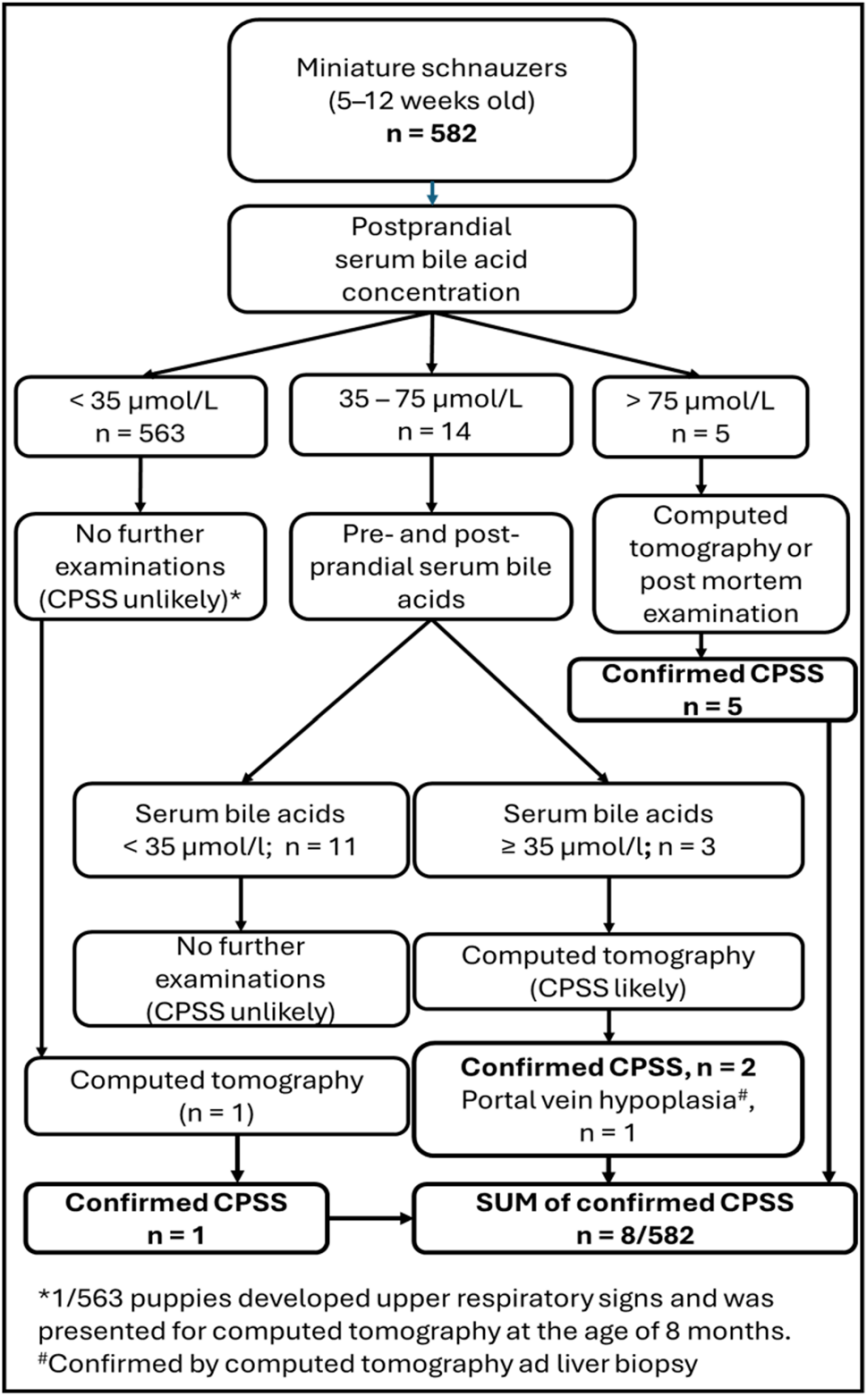
Flowchart of the congenital portosystemic shunt (CPSS) screening methods in miniature schnauzers

The interpretation of SBA results was based on reference values from YESLAB, the Central Laboratory of the Department of Equine and Small Animal Medicine, University of Helsinki, and previous literature [4, 18, 28]. A postprandial SBA concentration of < 35 µmol/L was considered normal. When the postprandial SBA concentration exceeded the upper reference limit (≥ 35 µmol/L), further investigations were recommended. Based on previous literature, postprandial SBA concentrations typically exceed 75 µmol/L in clinical cases of a CPSS [4]. If the postprandial SBA concentration was between 35 and 75 µmol/L, we recommended performing a complete SBA stimulation test approximately 1–4 weeks after the initial evaluation with pre- and postprandial sampling, as previously described. If the initial postprandial SBA concentration exceeded 75 µmol/L or, in the second SBA stimulation test, if the pre- and/or postprandial SBA concentrations were above the reference limits (≥ 20 µmol/L and ≥ 35 µmol/L, respectively), a CT scan was recommended. If the results of the second SBA test were within the reference limits, the puppy was assumed negative for a CPSS and excluded from further examinations. The diagnosis of a CPSS was confirmed by CT angiography or post-mortem examination. For CT scanning, a helical 64-slice multidetector CT scanner (LightSpeedTM VCT, GE Healthcare, Madison, WI, USA) was used. Post-mortem examinations were performed in the pathology unit of the Faculty of Veterinary Medicine, University of Helsinki. A flowchart of the screening and retesting is presented in Fig. 1.

### Laboratory analysis of serum bile acid

All the blood samples were assayed in YESLAB (Clinicum, Koetilantie 2, 00790 Helsinki, Finland). Samples from other veterinary clinics or hospitals were sent to the laboratory by mail within three days. The measurements were performed with a Konelab Prime 60i analyser (Thermo Fischer Scientific) using the Diazyme Total Bile Acid reagent (Daizyme Laboratories, Inc) to determine SBA concentrations. YESLAB has an external quality control scheme, and the laboratory runs daily quality control assessments. The remaining serum samples were identified, frozen and stored at -80 °C, enabling re-evaluation if needed.

### Statistical analyses

Descriptive statistics and calculation of the incidence of CPSSs were performed with SAS (System for Windows, version 9.4 SAS Institute Inc., USA). The Shapiro–Wilk test was used to assess whether the data followed a normal distribution. Descriptive statistics were presented as means (± SD) for continuous variables (age, SBA concentration) and as frequencies and the percentage distribution for categorical variables (sex and the location where the samples were taken: at VTHUH or another veterinary clinic or hospital). Incidences were calculated for both the one-year period during which the data were gathered and for the ten-year period, and 95% confidence intervals based on Wilson’s score were used to describe the precision of the estimated incidences.

## Results

### Signalments

The study population comprised 582/1004 puppies born in 121/216 litters and registered in Finland during the one-year study period from 2021 to 2022. The age of the puppies ranged from 5 weeks and 2 days to 11 weeks, and the mean (± SD) age was 50.9 (± 4.02) days for puppies without a CPSS and 51.3 (± 7.34) days for puppies with a CPSS.

Of the 582 puppies, 294 were females (50.5%). Descriptive statistics for age and sex according to the CPSS status are presented in Table 1.

**Table 1:** Descriptive statistics for miniature schnauzers according to the congenital portosystemic shunt (CPSS) status

Variable	Statistics	Bile acids in reference range (*n* = 574)	CPSS
Screening postprandial bile acid concentration	n	574	8
	Mean (SD)	13.0 (8.45)	72.7 (32.52)
	Median (Q1; Q3)	11.1 (7.5; 16.5)	83.5 (53.1; 96.4)
	Min, Max	0.8, 61.2	9.8, 105.8
Age of the dog (weeks)	n	574	8
	Mean (SD)	50.9 (4.02)	51.3 (7.34)
	Median (Q1; Q3)	51.0 (49.0; 52.0)	49.5 (47.0; 50.5)
	Min, Max	37.0, 76.0	47.0, 69.0
Sex			
F	n (%)	289 (50.3)	5 (62.5)
M	n (%)	285 (49.7)	3 (37.5)
Sample taken in			
Other	n (%)	300 (52.3)	8 (100.0)
VTHUH	n (%)	274 (47.7)	

Program: demog_v1mod.sas - Generated: 24MAY2024 1:18:59 PM

### Postprandial SBA concentrations

Of the screening blood samples, 47.1% (274/582) were taken at VTHUH and the others at cooperating veterinary clinics. Postprandial SBA concentrations of < 35 µmol/L were measured in 96.7% of the dogs (563/582) and ≥ 35 µmol/L in 3.3% (19/582), with five dogs having concentrations of > 75 µmol/L. Of the 14 dogs with SBA concentrations between 35 and 75 µmol/L at screening, 11 had pre- and postprandial SBA concentrations within the reference range (< 20 and < 35 µmol/L, respectively) 1–4 weeks after the first screening, and a CPSS was considered unlikely. No further investigations were performed for these dogs. In 3/14 dogs, either pre- or postprandial SBA concentrations were above the reference limit, and these dogs were subjected to further examinations. In one of these dogs, portal vein hypoplasia (PVH) was diagnosed by computed tomography and liver biopsy. The postprandial SBA concentration in this dog was 43.5 µmol/L.

Altogether, eight CPSS cases were diagnosed. Of these, five dogs had a screening postprandial SBA concentration exceeding 75 µmol/L and two dogs had concentrations of 45 µmol/L and 61.2 µmol/L, respectively. All these dogs were diagnosed with a CPSS by CT or post-mortem examination. One dog with a normal postprandial SBA concentration (9.8 µmol/L) was diagnosed with a CPSS 6 months later by computed tomography, which was performed due to clinical signs unrelated to the CPSS. For this dog, an additional complete SBA stimulation test was performed after the CPSS diagnosis, and the result was normal. Descriptive statistics for the bile acid concentration according to the CPSS status are presented in Table 1.

### Incidence of CPSSs

CPSS was detected in 8/582 dogs, giving a CPSS incidence of 1.37% in this study population. Extrapolating these data to the whole registered miniature schnauzer population of Finland, the one-year and ten-year incidences of CPSSs were both estimated to be 1.39% (Table 2).

**Table 2:** Incidence rates for congenital portosystemic shunts (CPSS) in miniature schnauzers

Data	Incidence %	Lower 95% CI	Upper 95% CI
Ten-year registered dogs (extrapolated)	1.39	1.17	1.66
One-year registered dogs (extrapolated)	1.39	0.83	2.33
Sample data	1.37	0.70	2.69

Program: prevalence.sas - Generated: 20JUN2024 3:59:45 PM

### CPSS morphology

A CPSS was diagnosed morphologically in all eight dogs: in five using computed tomography and in three following pathological examination. All shunts were single extrahepatic shunts inserting into the caudal vena cava at the level of the diaphragm. There was slight variation in the origin of the shunt; five of the shunts originated from the splenic vein and three from the left gastric vein. Two of the three dogs in which the CPSS originated from the left gastric vein were siblings from the same litter. The diameter of the shunts that were identified using CT varied between 4 and 6 mm, except for the one dog with a shunt as an incidental finding. In this dog, the diameter of the shunt was only 2 mm. The shunt originated from the splenic vein and inserted into the phrenic vein, eventually leading to the caudal vena cava.

## Discussion

We investigated the incidence of CPSSs in miniature schnauzer puppies to provide a possible estimate for the prevalence of CPSSs in this breed in Finland. This approach was based on the assumption that the prevalence of CPSSs must be at least equal to or greater than the incidence when the incidence is determined during the patients’ first months of life. Previous research on CPSSs in cairn and Yorkshire terriers has focused on estimating the prevalence [10, 11]. In our study, we expected to find an elevated incidence of CPSSs in miniature schnauzer puppies compared to most other breeds, and this assumption was correct in our study population. The incidence was lower than the prevalence of CPSSs in the general cairn terrier population in the Netherlands (2.5%) [11] and in the general Yorkshire terrier population in North America (3.6%) [10], but clearly higher than in general overall dog population (0.18–0.76%) [7, 13].

The late onset of diagnosis [12] and possible mild clinical signs in miniature schnauzers increase the risk of inadvertently breeding affected dogs. A screening test conducted as a puppy could greatly benefit miniature schnauzers. Early diagnosis allows for timely surgical intervention, potentially preventing long-term complications such as urolithiasis [29] and hepatic encephalopathy [6]. Moreover, it would enable breeders to make informed decisions to avoid using affected dogs for breeding.

Surgical attenuation generally provides better outcomes than conservative treatment alone, even for dogs diagnosed after five years of age [22, 30, 31]. However, an older age is a known risk factor for postoperative neurological complications [32]. A recent study [33] demonstrated that dogs over three years of age had a higher risk of developing postoperative seizures, with miniature schnauzers being the second most common breed in the study. Early diagnosis could reduce the risk of these complications.

In our study, it was of high importance that the screening method was easy to perform and accessible to ensure that as many breeders as possible would participate in the study and that the results could be considered representative for the whole population of the breed in Finland. We selected the postprandial SBA concentration as a screening method because of its reportedly high sensitivity and specificity [17, 18]. The test is easy to perform, as only one sample comprising a small amount of blood is needed. Moreover, the serum samples do not require special handling and can be sent elsewhere for analysis at room temperature. In comparison to the SBA stimulation test, with both pre- and postprandial sampling, postprandial SBA measurement is also faster, making it easier to schedule appointments with the breeders. These practical reasons were important, as we recruited participants throughout Finland.

Unfortunately, there are also some limitations when using only one screening method. The postprandial SBA concentration may vary due to several reasons: the emptying of the gallbladder may be early or incomplete, other liver diseases might be present, or the SBA result might be altered due to severe haemolysis or marked lipemia [17, 34]. In two studies, the sensitivity and specificity of postprandial SBA in detecting CPSS were both shown to be 100% [17, 18], and it is thus ideal for screening purposes. However, in a recent study [35] postprandial SBA had a sensitivity of 94.4% and a specificity of only 27.8%. In addition, Pena-Ramos et al. (2021) noted that 13.3% of dogs with hepatic vascular anomalies had postprandial SBA values below 30 µmol/L, questioning the value of postprandial SBA in ruling out a CPSS. However, the specific type of the hepatic vascular anomaly was not identified in nearly half of the dogs (31/64) within the group of congenital circulatory anomalies, which complicates the interpretation of the results [28]. It appears likely that in our study, some affected puppies might also have had a postprandial SBA concentration below 35 µmol/L. This was demonstrated for at least one dog with a small, 2-mm-diameter CPSS found incidentally in computed tomography that was performed because of clinical signs unrelated to the CPSS. The dog’s postprandial SBA concentration was 9.8 µmol/L during screening, and the dog did not display clinical signs related to a CPSS at the time of the diagnosis. This indicates that the CPSS had low clinical importance for this individual, but its presence remains still highly important when considering possible use of the dog in breeding. This finding also increases the probability that the incidence of CPSS may have been underestimated in our study population and the true prevalence of CPSS among miniature schnauzers can be higher.

Another option for mass screening would have been to measure the blood ammonia concentration. Unfortunately, ammonia is a very unstable metabolite that is prone to yielding false normal results if it is not analysed immediately or stored correctly [16]. Only a few veterinary clinics around Finland have in-house equipment for measuring the plasma ammonia concentration. In addition, other factors, such as diet, can affect ammonia levels [36], complicating the comparison of dogs with different nutritional backgrounds and ages, since the age of the litter also influences the composition of the puppies’ diet.

Twelve puppies with screening postprandial SBA concentrations above the reference limit were not diagnosed with a CPSS. One puppy was diagnosed with PVH after a repeated SBA increase, but the remaining eleven puppies were not investigated further after an SBA stimulation test yielded normal results. It is possible that some cases of vascular anomalies remained undetected. However, definitive exclusion would have required computed tomography under general anaesthesia and was not easily justified for puppies that appear clinically healthy. The possibility of undetected vascular anomalies could be further investigated in long-term follow-up studies on dogs that have been tested as puppies. The laboratory method may influence SBA concentrations, as shown in Maltese [34]. This study [34] reported higher SBA values using an enzymatic spectrophotometric method compared with high-performance liquid chromatography (HPLC). As the same method was used in our study, an analytical effect cannot be excluded; however, given that most values were below the reference limit, a similar effect to that observed in Maltese is considered unlikely [34].

Several similarities were observed among the eight study puppies that were diagnosed with a CPSS. All eight puppies were without clinical signs and there was no apparent reason to assume that these individuals would have a CPSS prior to screening. Abnormal postprandial SBA concentrations were measured in seven of the puppies in which a CPSS was confirmed, but one puppy had a normal postprandial SBA level. Interestingly, it is rather common that miniature schnauzers are diagnosed with a CPSS at an older age than dogs of other breeds and that the clinical signs can be mild when miniature schnauzers are puppies [12]. In our study, all the CPSS vessels inserted into the caudal vena cava at the level of the diaphragm. Previously, it has been speculated that the movement of the diaphragm can lower the amount of blood passing through the shunt if the insertion of the shunt is near the diaphragm, and this can lead to a higher amount of blood being led through the liver [37]. This could partly explain why the puppies had been without clinical signs, despite the CPSS, and why a CPSS can occur in part as an occult disease in miniature schnauzers. Based on these findings, it is highly important to thoroughly examine individuals that are intended to be used for breeding. A CPSS might not be detected early enough based solely on the clinical presentation in miniature schnauzers.

Our study did not include enough affected dogs to reliably assess heritability. However, the morphology of the shunts and incidence of the condition within the breed strongly suggests a genetic component. Further studies on heritability are needed in the future.

## Conclusions

The incidence of congenital portosystemic shunts in Finnish miniature schnauzers (1.39%) was elevated when compared to the estimated prevalence in the general dog population (0.18–0.76%). The morphology of the portosystemic shunts could indicate a common genetic background.

## Data Availability

The datasets used and/or analysed during the current study are available from the corresponding author upon reasonable request.
